# Aortobifemoral Bypass in Kidney Transplant Candidates: A Ten-Year Experience

**DOI:** 10.3389/ti.2024.12085

**Published:** 2024-02-06

**Authors:** Pascaline Bonnin, Salomé Kuntz, Sophie Caillard, Nabil Chakfé, Anne Lejay

**Affiliations:** ^1^ Department of Vascular Surgery and Kidney Transplantation, University Hospital of Strasbourg, Strasbourg, France; ^2^ Gepromed, Strasbourg, France; ^3^ Department of Nephrology and Kidney Transplantation, University Hospital of Strasbourg, Strasbourg, France

**Keywords:** kidney transplantation, vascular calcification, vascular surgical procedures, blood vessel prosthesis, kidney

## Abstract

In patients with severe aorto-iliac calcifications, vascular reconstructions can be performed in order to allow kidney transplantation. The aim of this study was to analyze the outcomes of kidney transplant candidates who underwent an aortobifemoral bypass (ABFB) for aorto-iliac calcifications. A retrospective study including all kidney transplant candidates who underwent an ABFB between 2012 and 2022 was performed. Primary outcome was 30-day morbidity-mortality after ABFB. Secondary outcome was accessibility to kidney transplant waiting list. Twenty-two ABFBs were performed: 10 ABFBs in asymptomatic patients presenting severe aorto-iliac circumferential calcifications without hemodynamic consequences, and 12 ABFBs in symptomatic patients in whom aorto-iliac calcifications were responsible for claudication or critical limb threatening ischemia. Overall 30-day mortality was 0%. Overall 30-day morbidity was 22.7%: 1 femoral hematoma and 1 retroperitoneal hematoma requiring surgical drainage in the asymptomatic group, and 2 digestive ischemia requiring bowel resection and 1 femoral hematoma requiring surgical drainage in the symptomatic group. Among the 22 patients, 20 patients could access to kidney waiting list and 8 patients underwent a kidney transplantation, including 3 living-donor transplantations. Aorto-iliac revascularization can be an option to overcome severe calcifications contraindicating kidney transplantation.

## Graphical Abstract

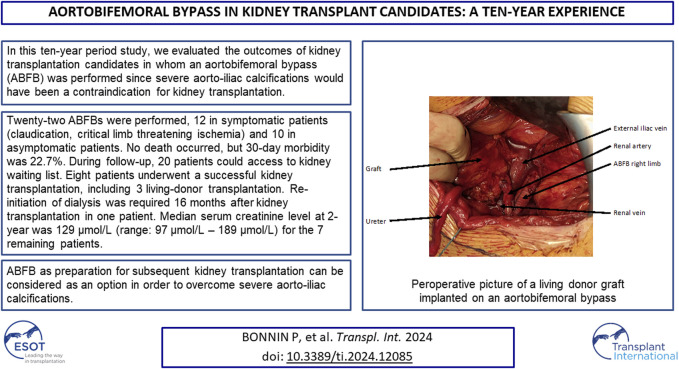

## Introduction

Aorto-iliac vascular disease is frequently found during the work-up for kidney transplantation due to increasing age and comorbidities accompanying end-stage renal disease. Moreover, it is well known that a positive association between calcification and age as well as time on dialysis exists [1, 2]. Accordingly, the number of kidney transplantation candidates presenting with aorto-iliac calcifications is increasing, due to the ageing of the population and the increasing time on dialysis before transplantation given organ shortage.

Severe calcification can cause a hemodynamically significant stenosis which needs repair in case its location is in the inflow tract of the kidney graft. On another hand, also in case of non-stenotic calcification, kidney transplantation can be contraindicated if there is no soft artery left for the clamping and arterial anastomosis. Accordingly, aorto-iliac calcifications are a common barrier to listing for kidney transplantation and among the patients on dialysis, around 33% of them are not on kidney transplant waiting list due to a vascular contraindication [3].

On another hand, kidney transplantation remains the best treatment modality for most patients with kidney failure to reduce all-cause mortality, but also regarding quality of life aspects and economic perspectives [4]. However, clear recommendations on the management of these patients with severe aorto-iliac calcifications are lacking. The Kidney Disease Improving Global Outcomes (KDIGO) guidelines state that aorto-iliac vascular disease is a relative contraindication for kidney transplantation but that selected patients can be considered for revascularization procedure to facilitate transplantation [5, 6].

Revascularization can include endovascular procedures such as iliac stenting, or perioperative iliac endarteriectomies in order to allow adequate clamping and arterial anastomosis. However, in some cases with severe and circumferential bilateral aorto-iliac calcification, a complete arterial reconstruction in order to prepare subsequent kidney transplantation is needed [7–9]. The aim of this study was to analyze the outcomes of kidney transplantation candidates who underwent aortobifemoral bypass (ABFB) for aorto-iliac calcifications over a 10-year period.

## Material and Methods

### Design of the Study

A retrospective review of a prospective database including kidney transplantation candidates who underwent an ABFB due to severe aorto-iliac calcifications between January 2012 and December 2022 was performed. The study was approved by the institutional review board. Severe aorto-iliac calcifications were defined as circumferential calcifications on both right and left iliac arteries not allowing arterial clamping and subsequent arterial anastomosis. Indications for ABFBs were discussed in multidisciplinary meetings including nephrologists, radiologists and vascular surgeons.

### Patient’s Data

The following preoperative parameters were recorded: demographic data (age, sex), cardiovascular risk factors (hypertension, diabetes mellitus, dyslipidemia, smoking, obesity), comorbidities (cardiac or pulmonary comorbidities), time on dialysis, and clinical presentation (asymptomatic, or symptomatic: claudication or critical limb threatening ischemia).

### Surgical Procedures

Revascularization data were recorded: operative time, blood loss, and need for transfusion. Length of intensive care unit was also recorded.

### Outcomes

Primary outcomes was defined as 30-day mortality and morbidity. Morbidity was defined as any digestive, ischemic or hemorrhagic complications.

Secondary outcomes was accessibility to kidney transplant waiting list. Post-transplant follow-up was recorded for patients who underwent kidney transplantation.

### Statistical Analysis

Not normally distributed data are presented with median value with data range (minimum to maximum).

## Results

During the study period, 22 ABFBs were performed for severe aorto-iliac in kidney transplantation candidates. Patients were 20 men and 2 women. Median age was 64 years (range 46–78 years).

All patients presented with hypertension, 7 were diabetics, 19 had dyslipidemia, 16 were former smoker, and 2 were obese. Median body mass index was 24.6 kg/m^2^ (range 18.9–33 kg/m^2^). Cardiac comorbidity (coronary surgery or stenting) was noticed in 8 patients and pulmonary comorbidity (chronic obstructive pulmonary disease) in 5 patients. Twenty patients were on hemodialysis (radiocephalic fistula in 9 patients, ulnar basilic fistula in 1 patient, brachiocephalic fistula in 8 patients, central venous catheter in 2 patients). Median time on dialysis was 2 years (range: 11 months–13 years).

Ten patients were asymptomatic with severe aorto-iliac circumferential calcifications without hemodynamic consequences, and 12 patients were symptomatic since aorto-iliac calcifications were responsible for claudication in 11 patients, and critical limb threatening ischemia with tissue loss in 1 patient. Among the 12 symptomatic patients, 1 patient also presented chronic mesenteric ischemia and a concomitant revascularization of the superior mesenteric artery was planned.

### Surgical Procedures

Median operating time was in average 3.5 h (range 2–6.2 h). Median blood loss was 690 mL (range 150 mL–2.1 L). Postoperative transfusion was required in six patients. Median length of Intensive Care Unit stay was 2 days (range 2–8 days).

### Primary Outcome

Thirty-day mortality was 0%. Overall 30-day morbidity was 22.7%: 1 femoral hematoma and 1 retroperitoneal hematoma requiring surgical drainage in the asymptomatic group, and 2 digestive ischemia requiring bowel resection and 1 femoral hematoma requiring surgical drainage in the symptomatic group.

### Secondary Outcome

**FIGURE 1:**
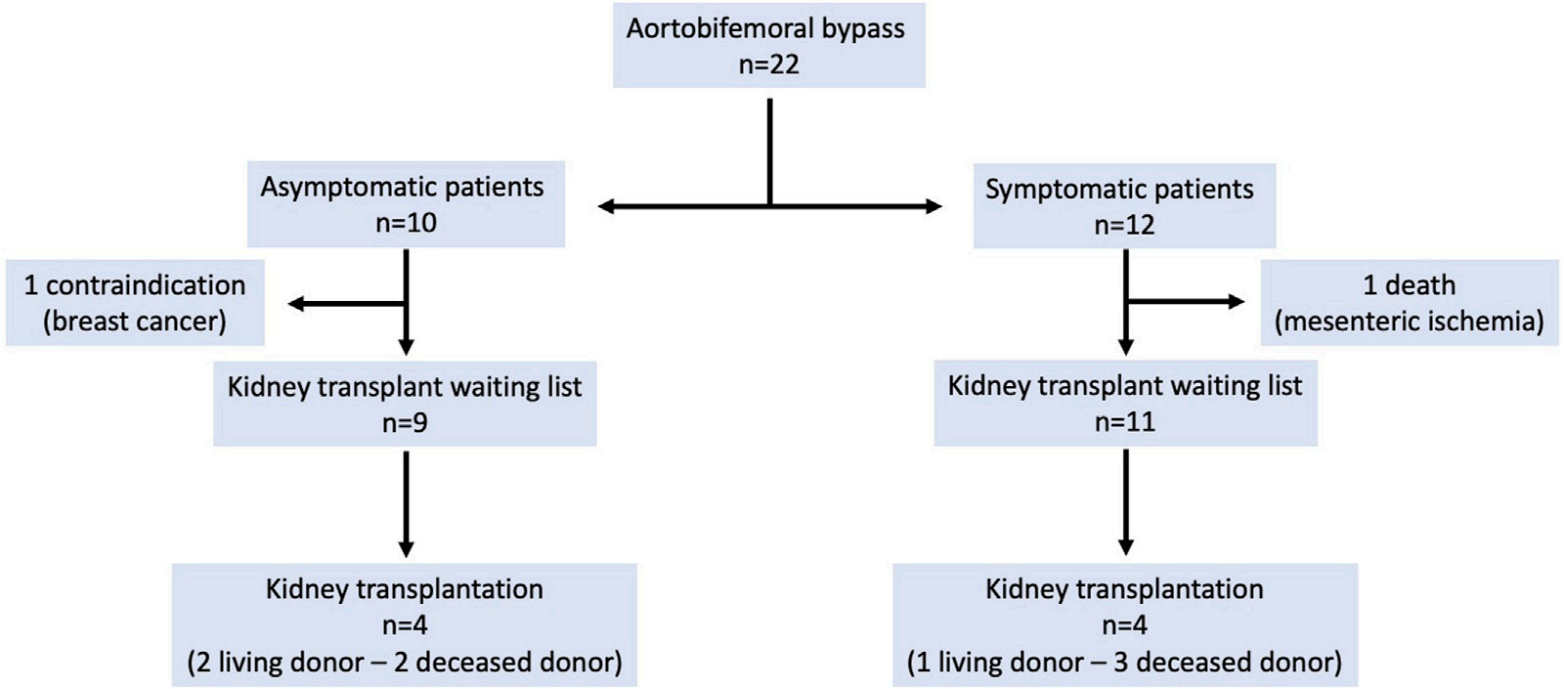
Outcomes of patients.

Follow-up of patients is presented in Figure 1. Median follow-up was 25 months (range: 2 months–8 years). The patient who underwent a concomitant revascularization of the superior mesenteric artery during ABFB died 15 months after the surgical procedure, from mesenteric ischemia. One patient was contraindicated since she was diagnosed a breast cancer. The 20 remaining patients could access to kidney waiting list. Median time between ABFB and registration (without contraindication) in kidney waiting list was 6 months (range 1–14 months).

**FIGURE 2:**
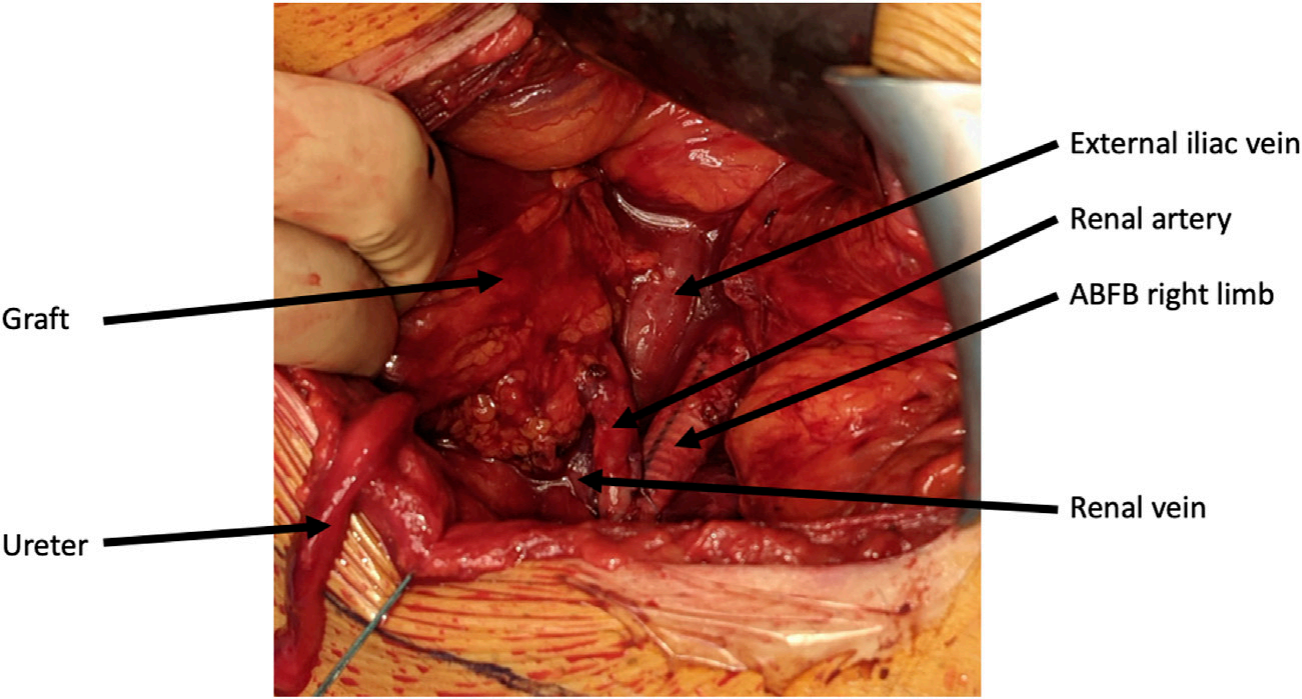
Per-operative picture of a living donor kidney graft implanted on an aorto-bi-femoral bypass.

Kidney transplantations were performed in 8 patients (Table 1): 5 deceased donor transplantations and 3 living donor transplantations (Figure 2). Median time between ABFB and kidney transplantation was 14 months (range: 5–63 months). Median operative time for kidney transplantation was 2.5 h (range 140–240 min) and median warm ischemia time was 23 min (range 24–44 min). Delayed graft function was noticed in 1 patient (patient 2), requiring dialysis for 1 week. Median length of hospital stay was 9 days (range: 6–13 days). Median serum creatinine level at discharge was 160 μmol/L (range: 101–307 μmol/L).

**TABLE 1:** Kidney transplantated patients.

Pedant	Age	Group	Time between ABFB and kidney transplantation	Donor	Donor age	Graft characteristics	Operative time (minutes)	Warm ischamia (minutes)	Serum aeatinine level at discharge (1 mol/L)	Serum creatinine level at 2-year (lnol/L)
1	63	Asymptomatic	10 months	Deceased donor	78	1 artery, 1 vein, 1 ureter	150	20	181	173
2	73	Asymptomatic	45 months	Dece donorased	83	1 artery, 1 vein, 1 ureter	150		189	307
3	53	Asymptomatic	6 months	Living donor	52	2 arteries, 2 veins, 1 ureter	240	44	132	107
4	70	Asymptomatic	10 months	Living donor	70	1 artery, 1 vein, 1 ureter	170	19	136	121
5	74	Symptomatic	18 months	Deceased donor	73	1 artery, 1 vein, 1 ureter	140	24	101	97
6	57	Symptomatic	5 months	Living donor	41	1 artery, 1 vein, 1 ureter	160	25	158	144
7	74	Symptomatic	63 months	Deceased donor	82	1 artery, 1 vein, 1 ureter	150	22	229	Hemodialysis
8	46	Symptomatic	48 months	Deceased donor	50	1 artery, 1 vein, 1 ureter	140	23	162	129

During follow-up, re-initiation of dialysis was required 16 months after kidney transplantation in one patient (patient 7). The patient presented polyomavirus associated nephropathy and acute cellular and humoral rejection. Median serum creatinine level at 2-year was 129 μmol/L (range: 97–189 μmol/L) for the 7 remaining patients.

## Discussion

Over a ten-year period, 22 ABFBs were performed in patients in whom severe aorto-iliac calcifications would have been a contraindication for kidney transplantation. Thirty-day morbidity was 22.7%. During follow-up, 20 patients could access to kidney waiting list and 8 patients underwent a successful kidney transplantation, including 3 living donor transplantation. Accordingly, ABFB as preparation for subsequent kidney transplantation can be considered as an option in order to overcome severe aorto-iliac calcifications.

However, an ABFP remains a high-risk procedure. Bredhal et al. reported the outcomes of 3,623 patients who underwent aortic surgeries for occlusive disease over a 20-year period: 30-day mortality was 3.6% and 30-day major complications rate was 20% [10]. In this study, renal insufficiency appeared as risk factor for 30-day mortality. Performing an ABFP in kidney transplant candidates is therefore risky, and it is mandatory to carefully select the patients susceptible to benefit from such a high-risk procedure. Moreover, the outcome of such transplantation is unpredictable, the expected patient survival can be low, such as the lifetime of the transplanted kidney. Undoubtedly, careful selection of patients is mandatory.

Performing an ABFB in symptomatic patients presenting with lower limb ischemia is less questionable, since revascularization is required in order to improve arterial insufficiency and therefore vascular-related symptoms, even in order to avoid major amputation. Revascularization in asymptomatic patients in whom aorto-iliac calcifications are not responsible for haemodynamic changes and vascular-related symptoms is however more questionable. Organ scarcity is global and the cost of transplantation including interventions for wait-listing is high. Accordingly, performing demanding surgery such as ABFBs in asymptomatic patients requires a careful selection and the decision whether or not to operate the patient must be based on a multidisciplinary discussion. Franquet et al. investigated the outcomes of 21 patients that underwent vascular bypass surgery prior to kidney transplantation without any vascular-related symptoms [11]. The authors reported that 2 patients (9.5%) died related to the bypass surgery and that early post-operative morbidity involved 11 patients (52.4%). Among the 21 patients, 11 (52.4%) were transplanted. Transplanted patients were significantly younger at the time of bypass and were less frequently treated for coronary heart disease. The authors concluded that aortic bypass surgery performed prior to kidney transplantation among asymptomatic patients has significant mortality and morbidity rates, but when transplantation is possible, results are satisfying. In our study, 10 ABFBs were performed in asymptomatic patients. No death occurred, but 30-day morbidity in this subgroup of patients was 20%. Four of the 10 asymptomatic patients were transplanted, the remaining patients are still on waiting list. In our experience, mortality and morbidity were lower than those reported by Franquet et al., but patients in our study might be younger with less comorbidities. Larger studies are therefore mandatory, in order to identify and better select patients in whom revascularization would benefit.

Open surgery has been the gold standard for revascularization procedures. With further advances in tools and techniques, endovascular procedures are increasing. It is obvious that percutaneous endovascular procedures should be the therapy of choice in kidney transplant recipients since they are less invasive, are associated with less morbidity and lower mortality, can be repeated if necessary and allow more rapid recovery of patients. However, in some patients with severe and circumferential bilateral aorto-iliac calcification, a complete arterial reconstruction in order to prepare subsequent kidney transplantation is required [8, 9]. Nevertheless, the timing of kidney transplantation is unpredictable and transplantation is not guaranteed even if revascularization is performed before. One might assume that revascularization, especially in asymptomatic patients, could be performed concomitantly to kidney transplantation. Gouny et al. reported the outcomes of five patients who underwent vascular procedures concomitant to kidney transplantation [7]. All patients had occlusive disease. An ABFB was performed in two symptomatic patients complaining from claudication. In two patients, aorto-iliac lesions were discovered intraoperatively and treated by iliac endarteriectomy. In the last patient, iliac lesions were initially neglected but an iliac endarteriectomy was necessary since the graft remained hypoperfused. One patient (patient with an ABFB) died at day 4 from septic shock and kidney rupture. The authors concluded that kidney transplantation is possible without major difficulties when ABFB is performed before surgery, but that severe complications are observed when kidney transplantation is performed concomitantly to revascularization procedures. Even if the advantages of simultaneous procedures are obvious, this strategy carries a significant risk of morbidity and mortality when a major surgery such as an ABFP is required. Accordingly, the authors recommended a two-stage procedure, with a minimal delay of 6 weeks between both procedures [12]. In our experience, a living donor kidney transplantation was planned in three patients. This could help selecting patients that could be considered for revascularization procedure to facilitate subsequent transplantation.

## Conclusion

ABFB as preparation for subsequent kidney transplantation can be considered as an option in order to overcome severe aorto-iliac calcifications. However, patients should be carefully selected and clear information should be given concerning morbidity and mortality. Further larger studies are however required in order to better identify the patients in whom such major revascularization procedures would benefit.

## Data Availability

The raw data supporting the conclusion of this article will be made available by the authors, without undue reservation.

## Abbreviations

ABFB, aortobifemoral bypass.

## References

[1] Blacher J Guerin AP Pannier B Marchais SJ London GM. Arterial Calcifications, Arterial Stiffness, and Cardiovascular Risk in End-Stage Renal Disease. Hypertension (2001) 38:938–42. 10.1161/hy1001.096358 11641313

[2] Kahn J Ram LM Eberhard K Groselj-Strele A Obermayer-Pietsch B Muller H. Calcification Score Evaluation in Patients Listed for Renal Transplantation. Clin Transpl (2017) 31:13. 10.1111/ctr.12888 27988970

[3] Vabret E Vigneau C Bayat S Frimat L É M Hannedouche T Who Are These Patients on Dialysis and Not on the Kidney Transplant Waiting List? Nephrol Ther (2020) 16:139–46. 10.1016/j.nephro.2020.02.014 32409290

[4] Chaudhry D Chaudhry A Peracha J Sharif A. Survival for Waitlisted Kidney Failure Patients Receiving Transplantation Versus Remaining on Waiting List: Systematic Review and Meta-Analysis. BMJ (2022) 376:e068769. 10.1136/bmj-2021-068769 35232772 PMC8886447

[5] Chadban SJ Ahn C Axelrod DA Foster BJ Kasiske BL Kher V KDIGO Clinical Practice Guideline on the Evaluation and Management of Candidates for Kidney Transplantation. Transplantation (2020) 104:S11–S103. 10.1097/TP.0000000000003136 32301874

[6] Rijkse E Kimenai HJAN Dor FJMF Ijzermans JNM Minnee RC. Screening, Management, and Acceptance of Patients With Aorto-Iliac Vascular Disease for Kidney Transplantation: A Survey Among 161 Transplant Surgeons. Eur Surg Res (2022) 63:77–84. 10.1159/000519208 34592735

[7] Gouny P Lenot B Decaix B Rondeau E Kitzis M Lacave R Aortoiliac Surgery and Kidney Transplantation. Ann Vasc Surg (1991) 5:26–31. 10.1007/BF02021773 1997072

[8] Nedelec M Glemain P Rigaud J Karam G Thuret R Badet L Renal Transplantation on Vascular Prosthesis. Prog Urol (2019) 29:603–11. 10.1016/j.purol.2019.06.005 31447181

[9] Galazka Z Grochowiecki T Jakimowicz T Kowalczewski M Szmidt J. Is Severe Atherosclerosis in the Aortoiliac Region a Contraindication for Kidney Transplantation? Transpl Proc (2011) 43:2908–10. 10.1016/j.transproceed.2011.08.023 21996186

[10] Bredahl K Jensen LP Schroeder TV Sillesen H Nielsen H Eiberg JP. Mortality and Complications After Aortic Bifurcated Bypass Procedures for Chronic Aortoiliac Occlusive Disease. J Vasc Surg (2015) 62:75–82. 10.1016/j.jvs.2015.02.025 26115920

[11] Franquet Q Terrier N Pirvu A Rambeaud JJ Long JA Janbon B Aortic Bypass Surgery for Asymptomatic Patients Awaiting a Kidney Transplant: A Word of Caution. Clin Transpl (2018) 32:e13218. 10.1111/ctr.13218 29394513

[12] Piquet P Berland Y Coulange C Olmer M Mercier C Rampal M. Aortoiliac Reconstruction and Renal Transplantation: Staged or Simultaneous. Ann Vasc Surg (1989) 3:251–6. 10.1016/S0890-5096(07)60034-X 2673318

